# A Cross-Sectional, Population-Based, Seroepidemiological Study of Rift Valley Fever in Cameroonian Cattle Populations

**DOI:** 10.3389/fvets.2022.897481

**Published:** 2022-06-14

**Authors:** Barend Mark Bronsvoort, Robert Francis Kelly, Emily Freeman, Rebecca Callaby, Jean Marc Bagninbom, Lucy Ndip, Ian Graham Handel, Vincent Ngwang Tanya, Kenton Lloyd Morgan, Victor Ngu Ngwa, Gianluigi Rossi, Charles K. Nfon, Stella Mazeri

**Affiliations:** ^1^Epidemiology, Economics and Risk Assessment (EERA) Group, The Roslin Institute, The Royal (Dick) School of Veterinary Studies, University of Edinburgh, Edinburgh, United Kingdom; ^2^Farm Animal Services, The Royal (Dick) School of Veterinary Studies, University of Edinburgh, Edinburgh, United Kingdom; ^3^School of Veterinary Medicine and Sciences, University of Ngaoundere, Ngaoundere, Cameroon; ^4^Laboratory of Emerging Infectious Diseases, University of Buea, Buea, Cameroon; ^5^Cameroon Academy of Sciences, Yaoundé, Cameroon; ^6^Institute of Ageing and Chronic Disease and School of Veterinary Science, University of Liverpool, Liverpool, United Kingdom; ^7^National Centre for Foreign Animal Disease, Winnipeg, MB, Canada

**Keywords:** Rift Valley fever (RVF), epidemiology, Cameroon, bovine, risk factor (RF)

## Abstract

Rift Valley fever (RVF) is an important emerging zoonoses causing abortion and neonatal deaths in livestock and hemorrhagic fever in humans. It is typically characterized by acute epidemics with abortion storms often preceding human disease and these events have been associated with the El Niño weather cycles. Outside of areas that experience epidemics, little is known about its epidemiology. Here, we present results from a serological study using biobank samples from a study of cattle conducted in 2013 at two sites in Cameroon. A total of 1,458 cattle from 100 herds were bled and sera screened using a commercially available RVF ELISA. The overall design-adjusted animal-level apparent seroprevalence of RVF exposure for the Northwest Region (NWR) of Cameroon was 6.5% (95% CI: 3.9–11.0) and for the Vina Division (VIN) of the Adamawa Region was 8.2% (95% CI: 6.2–11.0). The age-stratified serological results were also used to estimate the force of infection, and the age-independent estimates were 0.029 for the VIN and 0.024 for the NWR. The effective reproductive number was ~1.08. Increasing age and contact with wild antelope species were associated with an increased risk of seropositivity, while high altitudes and contact with buffalo were associated with a reduced risk of seropositivity. The serological patterns are more consistent with an endemical stability rather than the more typical epidemic patterns seen in East Africa. However, there is little surveillance in livestock for abortion storms or in humans with fevers in Cameroon, and it is, therefore, difficult to interpret these observations. There is an urgent need for an integrated One Health approach to understand the levels of human- and livestock-related clinical and asymptomatic disease and whether there is a need to implement interventions such as vaccination.

## Introduction

Rift Valley fever (RVF) is a zoonotic viral disease of ruminants, caused by a *Phlebovirus* in the *Bunyaviridae* family transmitted by floodwater mosquitos. It was first described in Kenya in 1931, and has since been reported in many countries in East and Southern Africa, as well as the Arabian Peninsula and sporadically in West Africa (1–3). It is considered an important emerging zoonotic pathogen of public health significance as its range is expanding, which is linked to climate change and expansion of the vector habitat. RVF affects mainly African communities with low resilience to economic and environmental challenges (4). Its epidemiology is characterized by explosive epidemics in both human and livestock populations, where livestock abortions can be a useful sentinel event for human disease risk (5). These events are often associated with flooding such as in El Niño years or dam construction and are followed by long inter-epidemic periods where there is little evidence of the viral presence in those populations affected by epidemics (6). RVF virus (RVFV) epidemiology of persistence during inter-epidemic periods as well as in areas without apparent epidemics is still poorly understood (4).

*Aedes* and *Culex* mosquitoes are the main vectors and potential reservoirs of the RVF virus (RVFV). The virus can be transferred vertically from female mosquitoes to their eggs in some species of the *Aedes* genera (7–9). Sheep, goats, and cattle are the domestic species most likely to be clinically affected but signs are usually mild or inapparent in adult animals. However, major outbreaks of abortions and death in neonates can occur during epidemic periods which result in significant direct economic losses (9–11). The virus can also infect wildlife species including Cape buffalo, as well as spillover into humans (12). The RVFV is transmitted between animals and humans through the bite of an infected mosquito vector. The disease in humans can also result from direct contact with infected tissues, blood, or body fluids (13). A rise in RVFV prevalence in domestic ruminants can sometimes precede epidemics in humans (5) and similarly a decline in herd immunity in the inter-epidemic periods coupled with extensive flooding appears to facilitate these explosive outbreaks (6). Symptoms of the disease in humans can vary, ranging from flu-like symptoms to more severe conditions such as meningoencephalitis, hemorrhagic fever, or death (9, 13). The case fatality rate for patients developing the hemorrhagic form of the disease can be as high as 50% (4).

Epidemiological studies of RVF have mostly focused on East Africa (14), where the virus was first isolated, with less information about its significance in Central and West Africa. However, outbreaks in human populations in Mauritania and Senegal have been associated with the development of new dam projects (4). In Central Africa, livestock exposure to RVF has been reported in the savanna of northern Cameroon [3–20% within small ruminant herds (2, 15, 16)] and Chad [4.4% in cattle, 10.7% in sheep, and 8.6% in goats (17)]. Most recently, in a large sample across Cameroon, seroprevalence estimates of 13.5% (11.4–15.7) for cattle and 3.4% (2.3–4.7) for small ruminants were reported (18).

Cameroon is a significant cattle producer in the Central-African region, with livestock contributing ~$476 million to the national economy in 2010 (19) and being of cultural importance to rural communities, particularly the pastoralist Fulani communities. The Northwest Region (NWR) and the Vina Division (VD) of the Adamawa Region of Cameroon are major cattle-keeping areas in the wider Adamawa Plateau of Central Africa. Cattle are kept for many reasons, including financial, draught power, dairy products, and trade. The area is mostly covered by sparse tree savannah, with a dry season between November and April, and a wet season from May until October (20). *Culex spp*. and *Aedes* spp. mosquitos are present in Cameroon (21) and due to the close association between cattle and people in the country, cattle may act as a reservoir for RVFV.

Despite the favorable climate for RVFV vectors and the abundance of livestock, little is known about the epidemiology of RVFV in Cameroon (18). This study aims to get population-based estimates of the seroprevalence of RVFV antibodies in cattle populations in two major cattle rearing regions of Cameroon and identify management or environmental factors associated with increased risk of individual animal-level seropositivity. In addition, we aim to estimate the force of infection and basic reproductive number.

## Materials and Methods

The authors have followed the STROBE (22) and recent STROBE-vet (23) guidelines for reporting observational epidemiological studies.

### Study Sites

**Figure 1:**
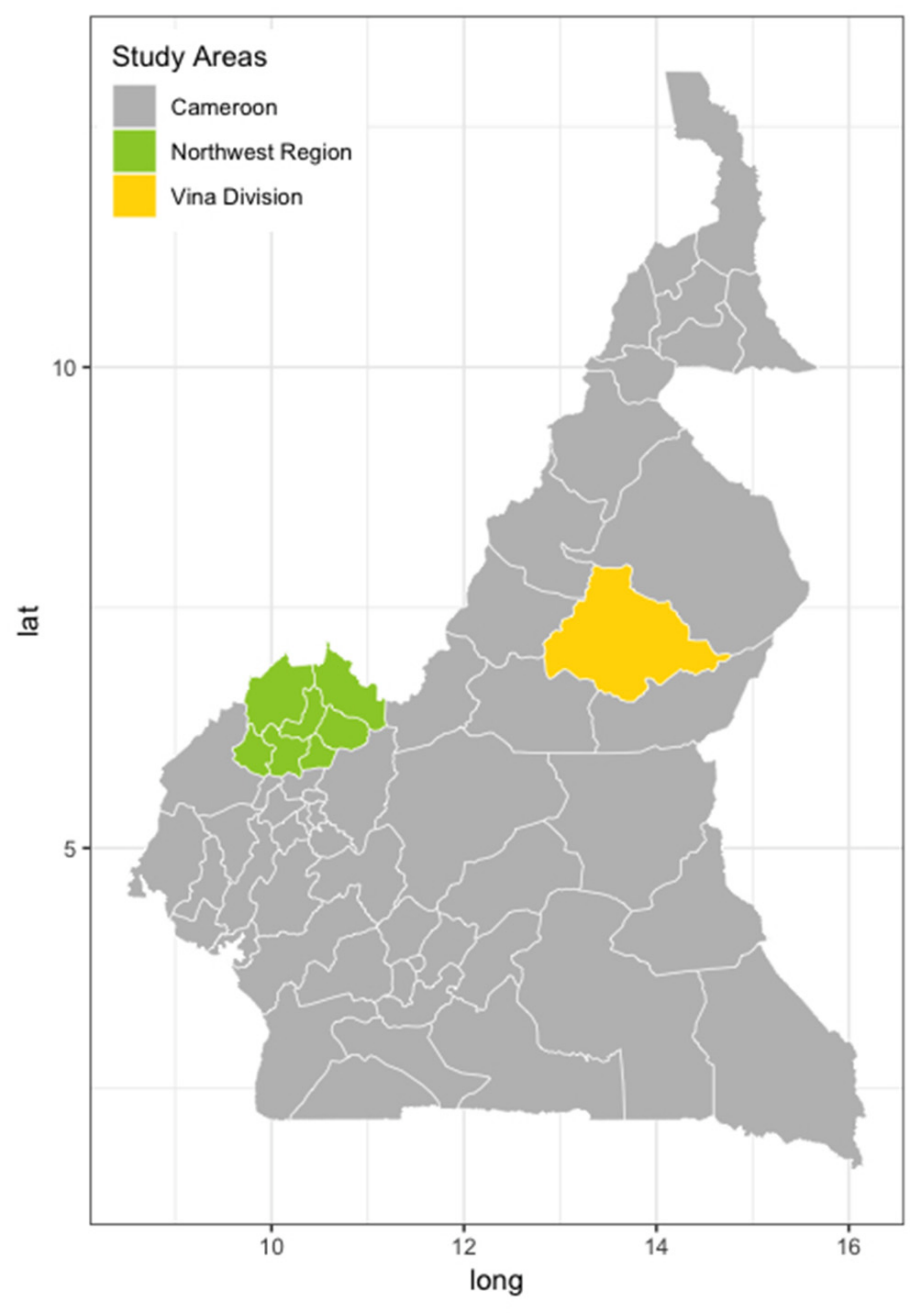
Map of Cameroon showing the two study locations. North West Region (green) and the Vina Division of the Adamawa Region (yellow) (24).

The study was conducted in two sites in Cameroon, one in the Northwest Region (NWR) and the other in the Vina Division (VIN) of the Adamawa Region (Figure 1). Both are of similar geographical size of ~17,000 km^2^. The NWR is situated in the fertile mountainous highlands, 500–3,000 m above sea level. Its regional capital, Bamenda, is Cameroon's third-largest city. The NWR is densely populated (1,804,695 people) and an estimated 506,548 cattle are grazed there (24). VIN is part of the fertile Adamawa Region's savannah plateau, and its regional capital is Ngaoundere. The population of the VIN (317,888 people) is much smaller than that of the NWR. The cattle population of VIN is also smaller with an estimated 176,257 herd (25). Veterinary services are predominately provided by the government through the Ministry of Livestock, Fisheries, and Industrial Agriculture/Ministere de l'Elevage des Peches et Industries Animales (MINEPIA), with local veterinary technicians stationed at Zootechnical and Veterinary Sanitary Control Centres (ZVSCC) distributed across the country roughly proportional to the local livestock population (20). Their responsibilities include registration of local livestock keepers, disease control mainly through annual vaccination campaigns, meat inspection, and regulation of livestock markets and animal movements.

### Study Design

A population-based stratified two-stage random cluster cross-sectional survey was conducted between January and May 2013 in the NWR and between September and November 2013 in the VIN. The participants were pastoralists whose herds were listed in the Ministry of Livestock, Fisheries, and Animal Industries vaccination records at 81 local veterinary centers in the NWR and 31 in the VIN in 2012. A total of 5,053 pastoralist herds in the NWR and 1927 in the VD, with a range of 1 to 215 cattle per herd were included in the sampling frame. The list of herds in each site was stratified by administrative area; there are seven divisions in the NWR and eight sub-divisions within the VIN. This gave roughly equivalent geographical areas for logistics and management purposes. A random sample of 50 herds was taken from each site and sampling was proportional to the total number of herds listed in each of the divisions/sub-divisions within each of the two sites. This survey was part of a larger study of bovine tuberculosis and liver fluke, and the sample size was based on a clustered random sample of cattle assuming a cattle level prevalence of ~10% (26), a within-herd variance of 0.15 and between herd variance of 0.01, an average herd size of 70, a relative sampling cost of 12:1 for herd: cattle, and relative error of ± 15% (Survey Toolbox; AusVet) (27). This gave a target sample size of 15 cattle per herd and 88 herds under the simplifying assumption of perfect test performance. To allow for potential losses or dropout and to have balanced samples from the two sites, we aimed for 50 herds in each of the two sites in the NWR and VIN. Within each herd, the 15 samples were stratified into three age classes; >6 months to <2 years old (young), 2–5 years old (adult), and older than 5 years (old).

### ID Screen^®^ Rift Valley Fever Competition Multi-Species ELISA

Several tests have been developed to detect RVF virus IgM and IgG antibodies in different species including a new commercial multi-species ELISA from ID. Vet (Montpellier, France) (28). The ID. Vet ELISA, which is easy to use in this setting, had an estimated overall diagnostic sensitivity (Se) of 0.854 (0.655–0.991 95% BCI) and specificity (Sp) of 0.986 (0.971–0.998 95% BCI) making it useful for surveillance activities or risk factor evaluation and reliable for evidence-based decision-making. The competitive ELISA was performed according to the instructions of the manufacturer and all the samples were run once. The plate was read at 450 nm. To control the validity of each plate, the mean value of the two negative controls (ODNC) was calculated and the plate was considered valid when OD_NC_ >0.7. For a valid plate, the mean value of the two positive controls divided by OD_NC_ should be <0.3. For each sample, the percentage positivity (PP) was calculated by dividing (OD_sample_/OD_NC_) x 100. The manufacturers suggest that if the value was ≤ 40%, the sample is considered positive. A value >50% was considered a negative result, and values between 40 and 50% indicated an inconclusive result. For the risk factor analysis, we used a single cut-off of ≤ 40% as positive and >40% as negative.

### Data Analysis

All data analyses and mapping were carried out using the R statistical language (29) version 4.0.4 within Rstudio (Boston, http://www.rstudio.com/) (Version 1.2.1335).

The population seroprevalence estimates were adjusted for the survey design with an animal-level and herd-level weighting. The animal-level weighting, *wa*_*j*_, was calculated as the number of animals sampled in a given herd j (na_j_), divided by the herd size (NA_j_), $\frac{na_{j}}{NA_{j}}$ and a herd-level weighting, *wh*_*k*_, was calculated as the number of herds sampled divided by the number of herds in the sampling data frame, $\frac{nh_{k}}{NH_{k}}$, for a given administrative division/sub-division. This gave the overall weighting for an animal of *wa*_*j*_×*wh*_*k*_. The *survey* package (30) was used to estimate the design-adjusted apparent seroprevalences within herds and at an administrative level and for the two sites assuming a perfect test. All estimates are given with 95% confidence intervals (CI).

Maps were generated using the *ggplot2* package (ref) and the shapefiles obtained from the open-access GADM database of Global Administrative Areas (www.gadm.org).

### Force of Infection λ and Effective Reproductive Number R_t_

The force of infection (FOI) λ is defined as “the rate at which susceptible individuals become infected per unit time,” or the probability that a susceptible individual will become infected per unit time and depends on the number of infectious individuals in a population and their contact rate with susceptible individuals. The force of infection λ was estimated using three methods for comparison using the Akaike's Information Criteria (AIC). A catalytic model first described by Muench that estimates a constant FOI was compared to two age-dependent models, one in which FOI is a linear function (Griffth's model) and a model in which FOI is a quadratic function of age groups (Grenfall-Anderson model). Generalized linear models were used as a statistical framework for each of the three models, adapted from Hens et al. (31). Data were organized into 1-year-wide age group except for animals over 10 years which were all grouped in a 5-year-wide band. The midpoint of each age category was used.

*R*_*t*_, the effective reproductive number, was estimated as follows: The average life expectancy was estimated as $\frac{1}{\mu }=\overset{\infty }{\underset{x=1}{\sum }}l_{x}$, where l_x_ is the survival rate at age x and is calculated as the ratio of the number of animals at age x divided by the number of animals in age class 1 (animals aged up to 1 year). For an individual of age *a*, the standard SIR (susceptible, infectious, recovered) model predicts that the probability that an individual is still susceptible is given by S(*a*) = exp [—*a*μ(*R*_t_ – 1)] under some simplifying assumptions, where *a* is the animal's age and μ is 1 over the average life expectancy. Since the numbers of the susceptible and infectious are binomially distributed, the likelihood function of these numbers was obtained as a function of *R*_t_. *R*_t_ was then inferred as the value that maximized the logarithm of this likelihood function:


$$\begin{array}{l} logL\left(R_{t}\right)=\sum {}_{i=0}^{n}\;\log\left(\exp\left(-a_{i}\mu \left(R_{t}-1\right)\right)\right)\;\\ \;+\;\sum {}_{i=0}^{m}\;\log(1-\exp\left(-b_{i}\mu \left(R_{t}-1\right)\right)) \end{array}$$

where *n* is the number of susceptible animals (seronegative) of age *a*_1_, …, *a*_*n*_ and *m* is the number of seropositive animals (ages *b*_1_, …, *b*_*m*_) for all farms (32).

### Mixed-Effects Multivariable Logistic Regression Modeling

At the time of sampling the herds, a questionnaire was also carried out with each herdsman. Several variables were identified as of interest and relevance for RVF. These included contact with wildlife (antelopes and buffalo), mixing with sheep and goats, breed, and contacts with other herds along with some key environmental factors including altitude, mean temperature, and different vegetation coverage (proportion of tree, shrubs, or grass), and distance to rivers or main roads.

Modeled climate data were downloaded from the UEA Climate Research Unit (version 4.03) (33). The mean temperature for the years 2011–2014 was extracted at the location of each georeferenced point. Landcover variables were downloaded from the European Space Agency (ESA) climate change initiative (CCI) landcover classification (34). To describe the landcover in the area surrounding the farm, the number of pixels of grassland, shrubland, and trees within 5 km of each point was extracted.

An initial screening of a small set of biologically relevant variables was carried out using univariable analysis. Variable selection was carried out using a forward stepwise approach. The multivariable mixed-effects logistic regression models were fit using the *glmer* function of package *lme4* with a binomial distribution and logit link (35) and the AIC was used to select the best model. The herd was included as a random effect to adjust estimates and precision for clustering within the herd. Age category and site (NWR/VIN) were also included to control for confounding.

## Results

**Figure 2:**
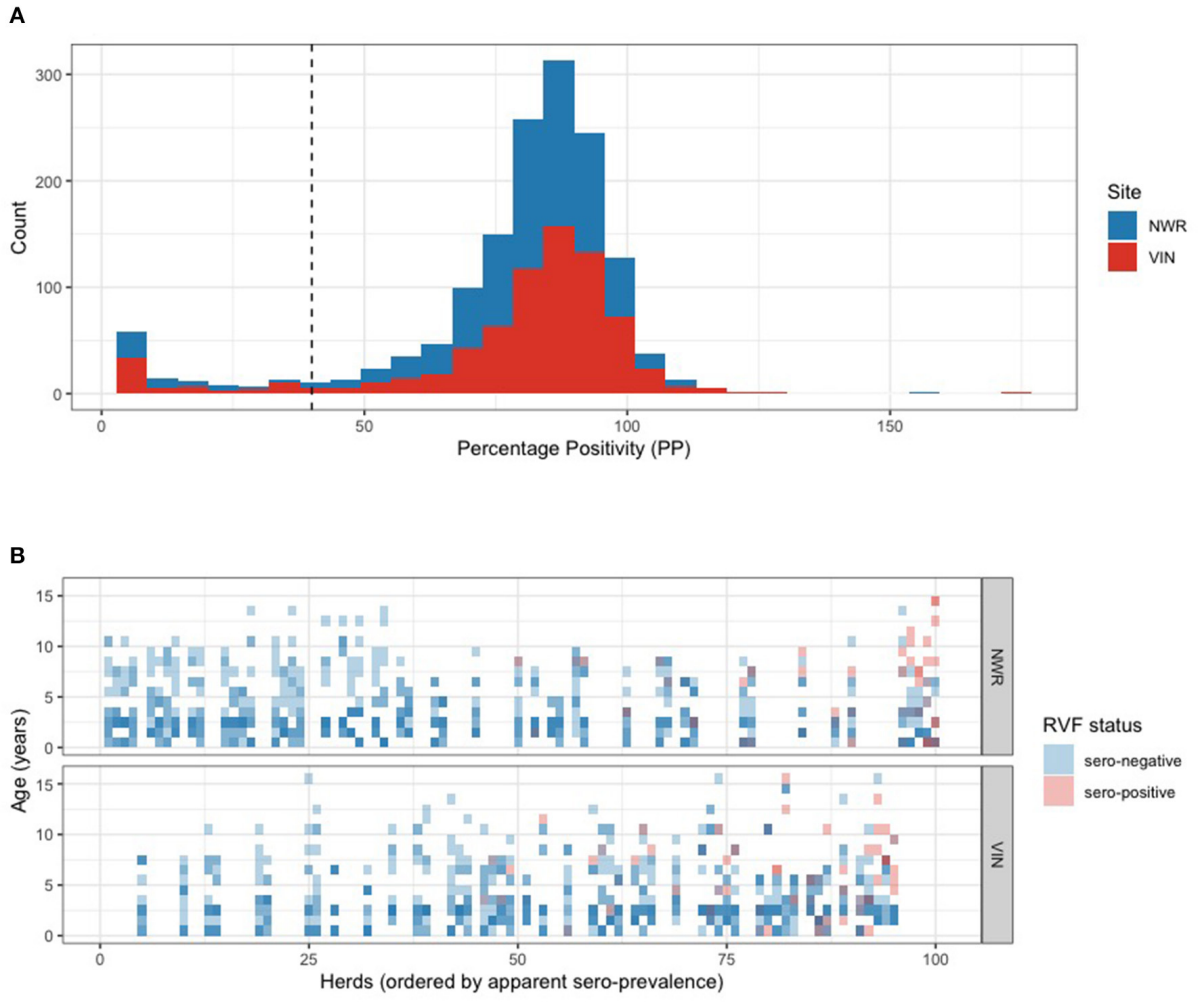
**(A)** Histogram of the percentage positivity results for individual cattle in Cameroon in 2012 for antibodies to RVFV using the ID.vet commercial multispecies ELISA. Cattle were considered seropositive if the percentage positivity (PP) value was ≤ 40. **(B)** Tile plot of individual animal seropositivity status, where seronegative animals are a blue tile and seropositive animals are a red tile (binary), by age in years (y-axis), grouped by herd (i.e., all the 15 animals from a herd are in the same vertical column), ordered by herd prevalence with lowest on the left to highest on the right (x-axis), and stratified by study site (Color opacity variation arises where tiles of more than one animal are overlaid).

A total of 1,498 cattle were sampled from 50 herds in each of the two study sites in Cameroon. The raw percentage positivity (PP) from the ELISA readings is plotted in Figure 2A and suggests a bimodal distribution. The majority (*n* = 1,381) of the animals were classed as seronegative with a distribution of PPs above the 40% manufacturers cut-off and a much smaller distribution of samples below 40% classed as positive. The individual binary (positive/negative) results are further plotted in Figure 2B. The vertical axis is age in years and the horizontal axis is herd rank-ordered by within-herd seroprevalence and stratified by the study site. This shows that more of the higher seroprevalence herds are in the VIN, although the highest six seroprevalence herds were in the NWR. The higher seroprevalence herds also have positives scattered across the ages.

**Figure 3:**
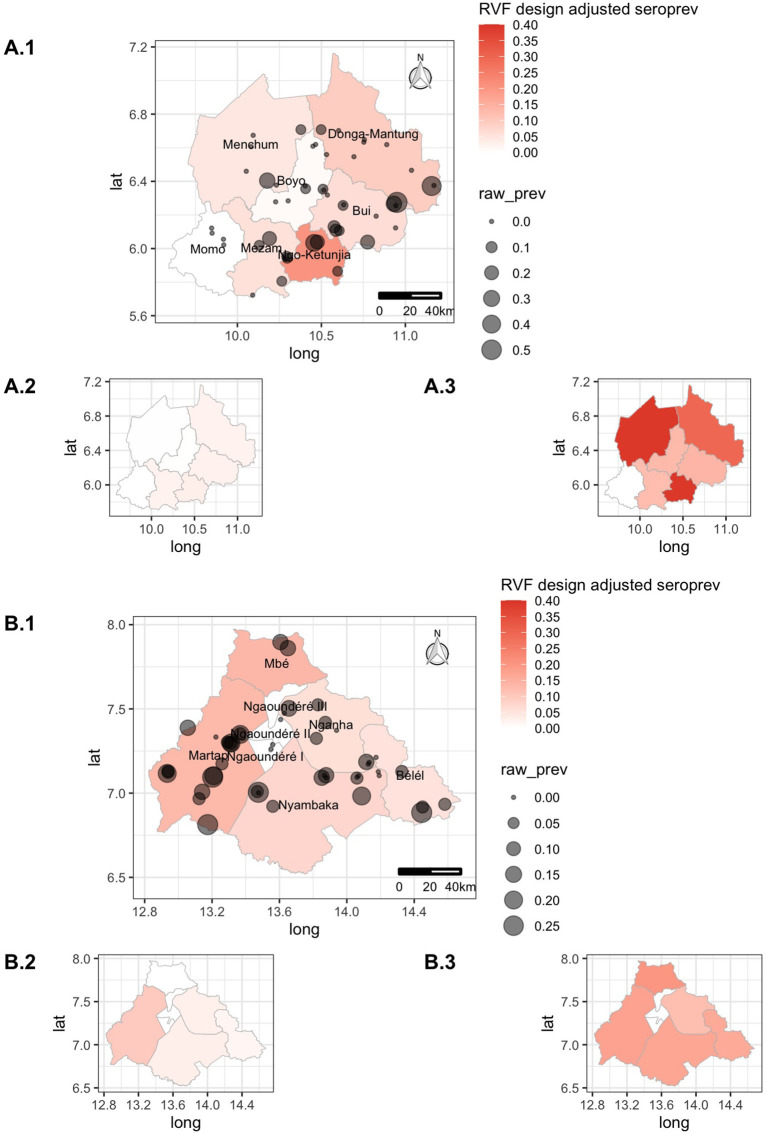
Choropleth maps of the Northwest Region (NWR) **(A)** and Vina Division (VIN) **(B)** in Cameroon colored by design-adjusted apparent seroprevalence for the administrative strata, overlaid with the approximate location of individual herds sized by the raw proportion of animals positive within each herd. The smaller inset choropleth maps are for the lower (X.2) and upper (X.3) 95% confidence intervals, respectively, for each site.

The proportion of seropositive herds (i.e., herds with >0 positive animals) was 42% (95% CI: 28.5–56.7) for the NWR and 68% (95% CI: 53.2–80.0) for the VIN and was statistically significantly different between the two sites (two-sample test for equality of proportions, *p*-value = 0.009). The animal-level seroprevalences per division/sub-division, adjusted for the sampling design, are presented in Table 1. The overall design adjusted animal-level seroprevalence of RVF for the NWR was 6.5% (95% CI: 3.9–11.0) and the VIN was 8.2% (95% CI: 6.2–11.0). Although the overall seroprevalences at the two sites were largely similar, there was considerable variation between the different administrative strata as can be seen in Table 1 and Figure 3. Ngoketunjia in the NWR and Mbé and Martap in the VIN had particularly high seroprevalences. Ngoketunjia includes a large dam project at its southern end but there are no obvious large water bodies that could simplistically explain higher seroprevalences in Mbé and Martap.

**Table 1:** Design-based animal-level seroprevalence (not adjusted for test performance) of RVF antibodies in cattle in Cameroon in 2013 stratified by division (NWR) and sub-division (VIN).

**Division/ sub-division**	**Raw proportions** **(positive/total)**	**Design-based** **seroprevalence**	**Design-based** **95% CI**
**North West region**			
Boyo	1/90	1.1	0.1–13.0
Bui	14/195	6.1	2.6–13.7
Donga -Mantung	17/180	9.3	2.4–29.7
Menchum	4/75	4.4	0.2–50.1
Mezam	7/105	5.4	2.3–12.1
Momo	0/60	0.0	0.0–0.0
Ngoketunjia	9/45	20.4	3.1–67.0
**Vina division**			
Belel	9/150	5.2	1.6–15.9
Martap	33/255	13.2	9.8–17.5
Mbe	4/30	13.3	0.0–20.0
Ngan-Ha	5/73	5.8	2.7–12.0
Ngaoundere	0/60	0.0	0.0–0.0
Nyambaka	14/180	7.5	3.1–16.8

**Figure 4:**
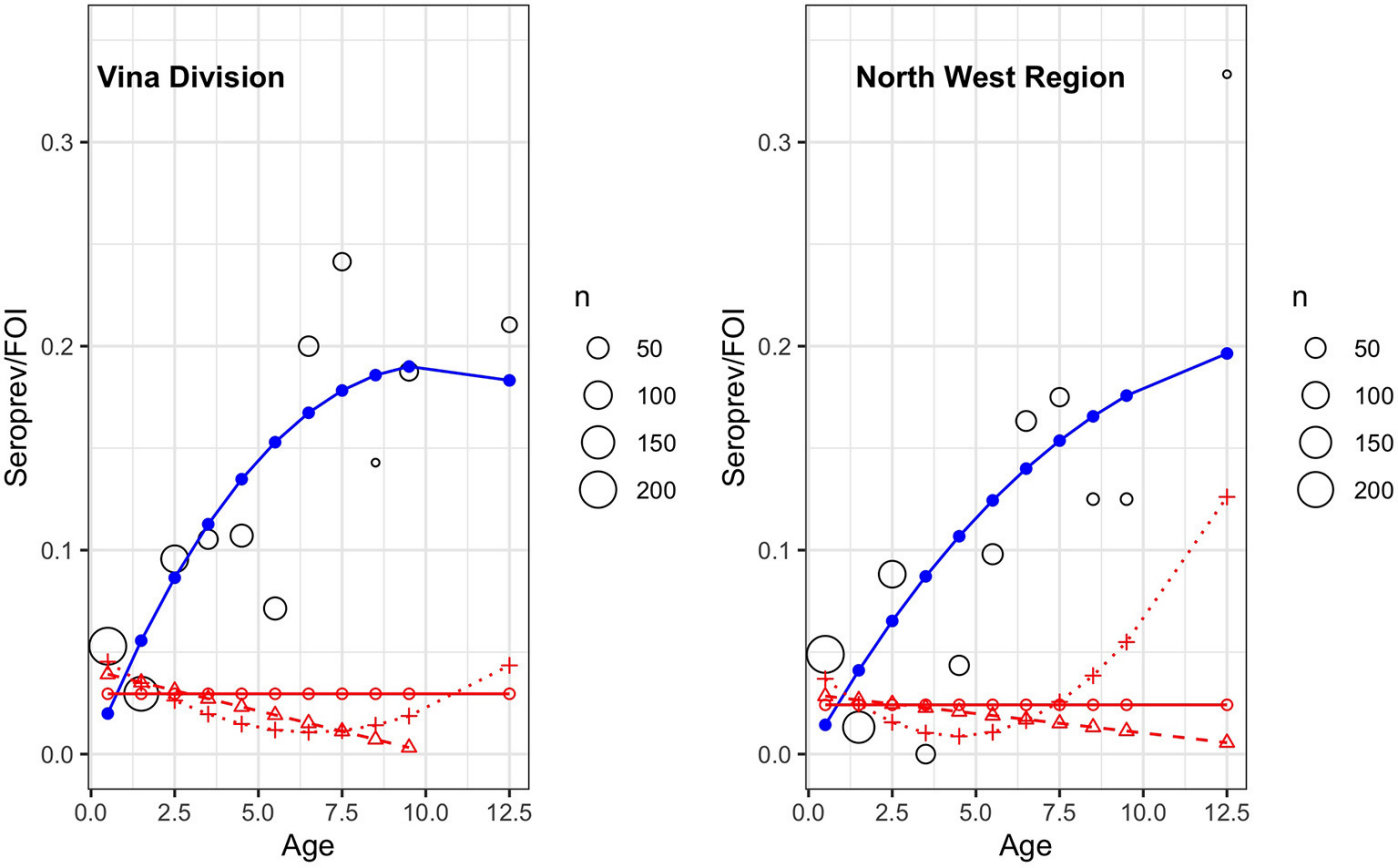
Age-stratified seroprevalence of RVF antibodies in cattle in two sites in Cameroon in 2012. The predicted seroprevalence based on a simple age quadratic function (blue) and the force of infection (FOI) (λ) based on the Muench (red circle/solid line), the Griffth's model (red triangle/dashed line), and the Grenfell Anderson model (red cross/dotted line). The black circles show the mean seroprevalence for that age strata with the size proportional to the number of animals in that age strata.

The age-stratified seroprevalence and the force of infection stratified by the site are plotted in Figure 4. As would be expected for an infectious disease like RVF, seroprevalence increases with age (and thus the time at risk of infection) and assumes that animals will remain seropositive after exposure. In both populations, the predicted seroprevalence does not appear to go above ~20%. The force of infection was modeled using three different models as shown in the various red lines in Figure 4. Their AICs are given in Table 2 and do not suggest that the more complex models are a markedly better fit for the data. The linear and quadratic models were the best for the VIN and NMR, respectively. The NWR model may be overly influenced by a small sample of older animals that had a particularly high seroprevalence but age-independent FOI is around ~0.029 for the VIN and ~0.024 for the NWR. Whichever model one chooses, the results suggest generally low FOI possibly declining with age. The FOI for VIN was slightly larger than for the NWR which may be related to the higher number of positive herds at this site. The effective reproductive rate R_t_ for the VIN was 1.10 compared to the NWR, where it was estimated as 1.08 suggesting little difference from a practical point of view.

**Table 2:** Force of infection (FOI) model comparisons for the VIN and NWR populations as plotted in Figure 4. Columns VIN and NWR contain the Akaike information criteria value (AIC) of model fit.

**Model**	**VIN**	**NWR**
Constant FOI (Muench' model)	59.082	64.063
Linear FOI (Griffth's model)	**56.889**	64.958
Quadratic FOI (Grenfell-anderson model)	57.560	**62.369**

*For each fitted model, with lowest AIC per site in bold*.

**Figure 5:**
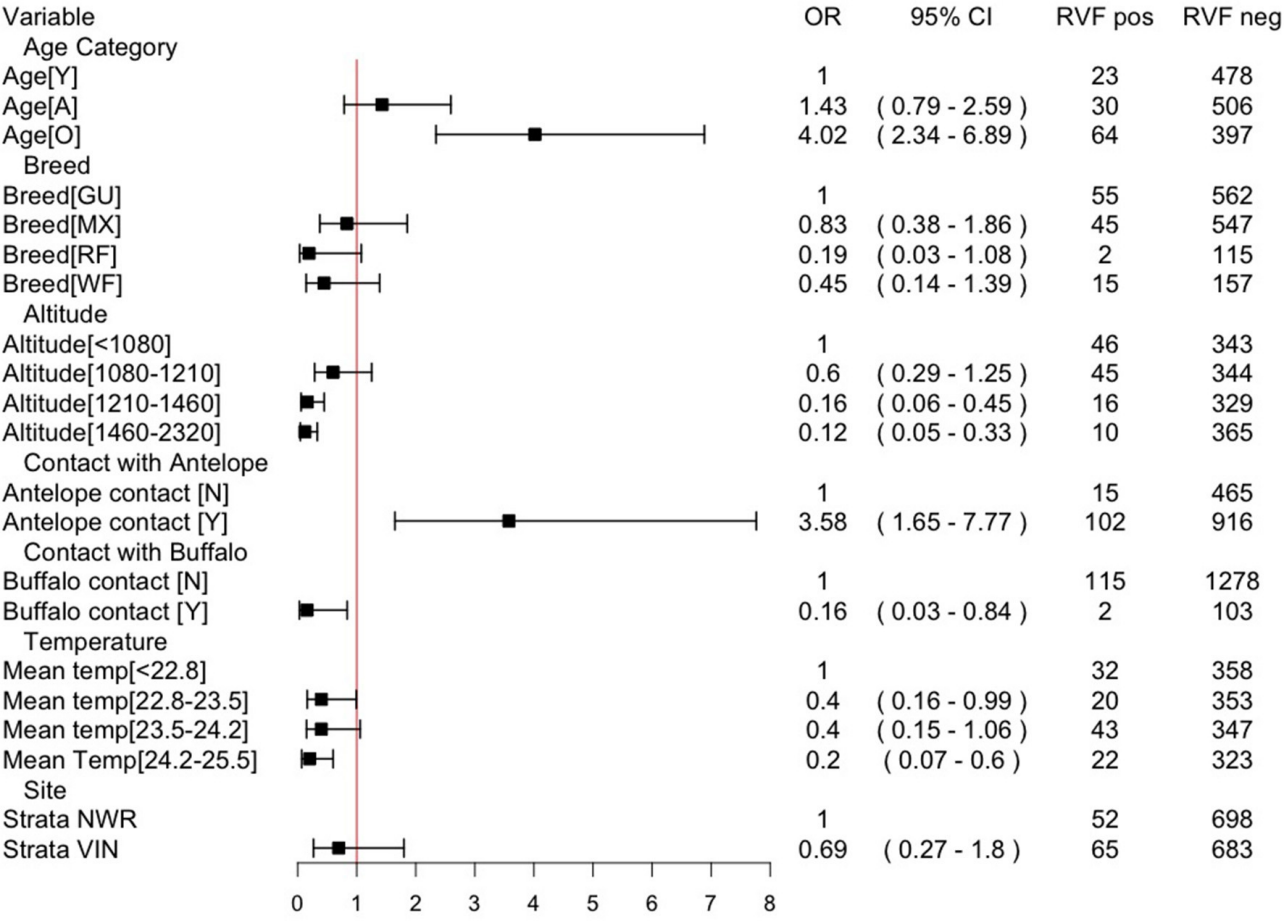
Final multivariable mixed effects logistic regression model, with herd as the random effect, for animal-level seropositivity for RVF in cattle in Cameroon in 2013. Intra-cluster correlation coefficient (ICC) = 0.13; residual error = 3.29; herd-level variance = 0.48; marginal *R*^2^ = 0.323; conditional *R*^2^ = 0.409; Area Under the Curve (AUC) = 0.785. NB Y= young (<2 years); A, adult (2–5 years); O, old adult (> 5years); GU, Gudali; MX, mixed breed; RF, Red Fulani; WF, White Fulani; OR, odds ratio.

A mixed-effects multivariable logistic regression model was developed to explore the association of various risk factors with seropositivity at the animal-level adjusting for clustering by the herd. The model-building strategy is given in Table 3. The odds ratios for the final model are presented in Figure 5. The risk of being seropositive increased with age (as would be expected) and also with contact with antelopes (though the exact species were not known by the herdsmen). In contrast, the odds of seropositivity decreased with higher altitude and higher temperatures. It also decreased with contact with buffalo suggesting a complex relationship with wildlife contacts. Finally, the administrative strata were included as a fixed effect, but there is little supporting statistical evidence that there is a residual strata difference. The intra-cluster correlation is relatively small at 0.13 suggesting that most of the variance is at the animal rather than the herd level. The marginal *R*^2^ (the variance explained by the fixed effects) is ~32% and the conditional *R*^2^ (the variance explained by both the fixed and random effects) is ~41%, with an AUC of ~78% suggesting the model is useful and explains a reasonable amount of the variation.

**Table 3:** Stepwise forward selection of final hierarchical multivariable logistic regression model (with herd fitted as a random effect).

**Model**	**AIC**
rvf_PN40 ~ (HER_ID)	786.9
rvf_PN40 ~ AGE2 + (HER_ID)	757.9
rvf_PN40 ~ AGE2 + strata1 + (HER_ID)	757.9
rvf_PN40 ~ AGE2 + GPSALT + (HER_ID)	744.4
rvf_PN40 ~ AGE2 + GPSALT + ANTEVR + (HER_ID)	739.2
rvf_PN40 ~ AGE2 + GPSALT + ANTEVR + BUFEVR + (HER_ID)	732.2
rvf_PN40 ~ AGE2 + GPSALT + ANTEVR + BUFEVR + dist2rdmain + (HER_ID)	732.6
rvf_PN40 ~ AGE2 + GPSALT + ANTEVR + BUFEVR + dist2rdmain + strata1 + (HER_ID)	729.9
**rvf_PN40 ~AGE2+ABREED+GPSALT+ANTEVR+BUFEVR+MeanTemp+strata1+(HER_ID)**	**726.5**
rvf_PN40 ~ AGE2 + ABREED + GPSALT + ANTEVR + BUFEVR + MeanTemp + dist2rdmain + strata1 + (HER_ID)	728.4
rvf_PN40 ~ AGE2 + GPSALT + ANTEVR + BUFEVR + strata1 + (HER_ID)	732.7
rvf_PN40 ~ AGE2 + GPSALT + ANTEVR + BUFEVR + MeanTemp + strata1 + (HER_ID)	727.1
rvf_PN40 ~ AGE2 + GPSALT + ANTEVR + BUFEVR + MeanTemp + strata1 + shrubs +Tree +Grass + (HER_ID)	731.7

*N.B. rvf The tables shows the different models and their corresponding AIC value. _PN40, “RVF status as positive/negative”; HER_ID, “Unique herd identifier,” fitted as the random effect; AGE2, “Categorical age with three levels”; GPSALT, “altitude in meters as measured by handheld GPS”; ANTEVR, “Does the herd have contact with antelope when on transhumance of where they normally graze—yes/no”; BUFFEVR, “Does the herd have contact with wild buffalo when on transhumance of where they normally graze—yes/no”; dist2rdmain, “distance to a main road”; MeanTemp, “mean annual temperature”; strata1, “Site—NWR/VIN”; Shrubs, tree, and grass are the number of pixels of each landcover type form the ESA landcover classification (resolution 20 × 20 m) within a 5-km radius of the GPS location of the herd*.

## Discussion

This study is the first population-based livestock study of RVF in Cameroon to estimate the effective reproductive rate R_t_, the FOI, and identify risk factors for RVF seropositivity in cattle in the country. It highlights that there is a high seroprevalence in the cattle population and therefore may be an important differential for abortion in cattle (and other small ruminants or camels). The estimates for the VIN, 68% (95% CI: 53.2–80.0), and NWR, 42% (95% CI: 28.5–56.7), are similar to those reported by Rissmann et al. (18) but are not directly comparable as their estimates do not separate species or adjust for population sizes and sampling. However, that RVF has now been reported in small ruminants and cattle by several studies also emphasizes its importance as a zoonoses, particularly, for herdsmen, women and children, and abattoir workers. A meta-analysis by Nicholas et al. (36) identified several specific human risk factors including being male, handling aborted animal tissues, helping with birthing, skinning an animal, slaughtering an animal, and drinking raw milk. These are all activities many herdsmen and slaughterhouse workers as well as wider family members of herdsmen are likely to be carrying out and therefore RVF needs to be on the differential lists of clinicians. In addition, the infected cattle population will be acting as a major reservoir of infection for the region. Interestingly, RVF is not widely reported in humans in Cameroon and they do not appear to currently suffer the large abortion storms in ruminants seen in East and North Africa. This may in part be explained by the lack of reporting due to the remote nature of the setting and lack of access to health or veterinary care. It may also suggest that there is something different about its epidemiology in the region or may reflect the lack of diagnostic effort or both. Anecdotal evidence from discussions with local clinicians highlighted that a high proportion of cases in hospitals are fevers of unknown origin that tend to be undiagnosed. A recent small study of African rainforest hunter-gatherers, known as the Baka ethnic group, in Eastern Cameroon reported a 12.4% seropositivity in this population (37). However, there needs to be much more comprehensive surveillance and diagnostic capability in Cameroon and West and Central Africa, generally so that both veterinary and human clinical cases can be identified and correctly managed.

Infection appears to be widespread across both the NWR and VIN with some evidence of hotspots such as Ngoketunjia, Mbé, and Martap. The overall seroprevalence appears to be <10% in these two populations compared to high clinical incidence areas in South Africa where they report 42% seropositivity in cattle (38). The age-independent FOI (the probability an individual becomes infected in a given year) estimates of ~0.029 for the VIN and ~0.024 for the NWR are in line with other estimates in cattle in Madagascar (39) and give a useful starting value for future modeling. However, the best fitting models suggest a more complex relationship with FOI declining slightly with age.

Entomological risk factors include temperature, rainfall, and biotic factors such as breeding sites and vertebrate hosts. Cameroon has plenty of potential vertebrate hosts and high seasonal rainfall in the areas studied, although typically you do not tend to see the flooded pans described in East and Southern Africa. There is little in the literature about the species competence of mosquitoes in Cameroon to transmit RVF. Simonet et al. (21) looked at Northern Cameroon and identified 9 primary and 22 secondary vector species capable of transmitting RVFV, and the authors highlight the need for PCR analyses of potential vectors to understand their true importance.

The role of wildlife may also be important. Here, we observed a very strong association with increased risk when herds reported contact with antelope, but a reduced risk when they reported contact with buffalo. Only antelope may be a reservoir in these areas and buffalo are currently not infected. Several studies have reported high seroprevalences in wildlife species including various antelopes and buffalo but their importance is less clear from an epidemiological point of view (40). In this Kenyan study, seroprevalence was especially high in some wildlife species. There may be vector-feeding preferences linked to species of wildlife and how they interact with livestock that we currently do not understand. In Cameroon, there are increasing conflicts between pastoralists and crop farmers (41), and this is changing patterns of grazing and transhumance that will impact transmission potential with wildlife.

Much of the variation is explained by altitude, with lower altitude areas having a higher risk, but this is countered by a reduced risk at higher temperatures suggesting a complex environmental interaction presumably driving mosquito breeding and feeding habits and virus replication and amplification within the vector. The age-stratified analysis suggests a more stable endemic epidemiological pattern rather than a more periodic epidemic outbreaks pattern. Although correlation between RVF outbreaks and the warm phase of El Niño/Southern Oscillation (ENSO) phenomena which lead to abnormal rainfall has been reported (7), there have been instances where no outbreaks were recorded following seasons of exceptionally above normal rainfall (42). Moreover, in some sub-Saharan regions, such as West Africa, RVF outbreaks are not known to be correlated with above-average rainfall (43). The lack of reports of either livestock or human outbreaks and the R_t_ of ~1.08 as well as the clear pattern of increasing risk of exposure with age may mean that there is a different epidemiological process and a more stable low impact endemic situation in Cameroon possibly related to a different climatic cycle less influenced by El Niño. This study has focused on cattle because a sera biobank was available for screening. However, given the high seroprevalence of RVF as well as other zoonoses such as Congo-Crimea hemorrhagic fever (44), brucellosis leptospirosis, and Q fever (45), there is an urgent need for a more One Health-based approach looking across species as well as human populations to understand the real impacts of these diseases.

## Conclusions

RVF virus appears to be circulating widely in the livestock rearing areas of Cameroon. However, there are no reports of livestock abortion storms or human clinical disease, though this may be due to limited investigations conducted in the country. The serological results suggest a more endemic epidemiological pattern that may be different from the cyclical epidemics seen in East Africa linked to El Niño. There is an urgent need for One Health-based study to understand the clinical scale and impacts of the RVF virus both on the livestock and human populations in Cameroon to direct interventions to reduce disease burden and antimicrobial misuse.

## Data Availability Statement

An anonymised version of the data included in the article is available on request to the corresponding author.

## Ethics Statement

The animal study was reviewed and approved by the Institute of Research and Development (Cameroon) and the University of Edinburgh Ethics Committee (UK) approved the study at the moment of data collection (VERC No: OS02-13). Written informed consent for participation was not obtained from the owners because verbal permission was obtained from all herdsmen in order to collect the biological samples from the animals and before administering the questionnaire. A brief explanation of the purpose and procedures of the study preceded the consent and herdsmen were informed of the possibility of opting out at any stage. Verbal consent was used as most herdsmen had limited formal education.

## Author Contributions

BB, RK, KM, VNT, and LN conceived and designed the project. RK collected the data. RK, JB, and VNN processed samples and conducted ELISA tests. EF, RC, SM, and BB conducted the data cleaning and analysis. BB, EF, and JB drafted the manuscripts. All authors contributed to the article and approved the submitted version.

## Funding

BB acknowledges the support provided by the Wellcome Trust [WT094945]; this grant funded the original fieldwork. BB is supported by BBSRC Institutional funding (BBS/E/D/30002275). The funders had no role in study design, data collection and analysis, decision to publish, or preparation of the manuscript. Importantly we would like to thank all the cattle keepers, MINEPIA veterinary centre staff and delegates whom without this study would not possible.

## Conflict of Interest

The authors declare that the research was conducted in the absence of any commercial or financial relationships that could be construed as a potential conflict of interest.

## Publisher's Note

All claims expressed in this article are solely those of the authors and do not necessarily represent those of their affiliated organizations, or those of the publisher, the editors and the reviewers. Any product that may be evaluated in this article, or claim that may be made by its manufacturer, is not guaranteed or endorsed by the publisher.
